# Rotavirus and Norovirus Infections in Children Under 5 Years Old with Acute Gastroenteritis in Southwestern China, 2018–2020

**DOI:** 10.1007/s44197-022-00050-8

**Published:** 2022-07-20

**Authors:** Longyu Yang, Shulan Shi, Chen Na, Bai Li, Zhimei Zhao, Tao Yang, Yufeng Yao

**Affiliations:** 1 Yunnan Key Laboratory of Vaccine Research & Development On Severe Infectious Disease, Department of Immunogenetics, Institute of Medical Biology, Chinese Academy of Medical Sciences and Peking Union Medical College, Kunming, 650118 Yunnan People’s Republic of China; 2 Institute of Pediatrics, Children’s Hospital Affiliated to Kunming Medical University, Kunming, Yunnan 650228 People’s Republic of China; 3 Department of Pediatrics, Yanan Hospital Affiliated to Kunming Medical University, Kunming, Yunnan 650000 People’s Republic of China

**Keywords:** Rotavirus, Norovirus, Pediatric acute gastroenteritis, Epidemiology, Southwestern China

## Abstract

**Objective:**

Rotaviruses and noroviruses are important causes of acute gastroenteritis in children. While previous studies in China have mainly focused on rotavirus, we investigated the incidence of norovirus in addition to rotavirus in Southwestern China.

**Methods:**

From January 2018 to December 2020, cases of rotavirus or norovirus infections among children under five ages with acute gastroenteritis were evaluated retrospectively.

**Results:**

The detection rate of rotavirus was 24.5% (27,237/111,070) and norovirus was 26.1% (4649/17,797). Among 17,113 cases submitted for dual testing of both rotavirus and norovirus, mixed rotavirus/norovirus infections were detected in 5.0% (859/17,113) of cases. While there was no difference in norovirus incidence in outpatient compared to hospitalized cases, rotavirus was detected two times more in outpatients compared to hospitalized cases (26.6% vs.13.6%; *P* < 0.001). Both rotavirus and norovirus infections peaked in children aged 12–18 months seeking medical care with acute gastroenteritis (35.6% rotavirus cases; 8439/23,728 and 32.5% norovirus cases; 1660/5107). Rotavirus infections were frequent between December and March of each year while norovirus was detected earlier from October to December. Our results showed significant correlation between virus detection and environmental factors such as average monthly temperature but not relative humidity. In addition, we observed a reduction in the detection rates of rotavirus and norovirus at the beginning of the SARS-CoV-2 pandemic in 2020.

**Conclusion:**

Our results indicate that rotavirus and norovirus are still important viral agents in pediatric acute gastroenteritis in Southwestern China.

## Introduction

Acute gastroenteritis (AGE) caused by bacteria, viruses and parasites is the second leading cause of morbidity and the most important cause of malnutrition among children under 5 years old globally, posing a heavy burden to the economy and public health [1]. In China, according to the National Notifiable Diseases Reporting System, infectious diarrhea (excluding cholera, dysentery, and enteric fever) has been classified as a legal Class C infectious disease and the second leading notifiable disease; more than one million cases were reported annually from 2018 to 2020 [2–4].

With improvements in living standards, sanitation, water treatment and food safety awareness, enteric viruses have replaced bacteria as the most significant pathogen of AGE. Of these viruses, rotavirus and norovirus have been recognized as the major pathogens of pediatric viral gastroenteritis [5].

Rotaviruses (RoVs) are non-enveloped particles with a genome consisting of 11 segments of double-stranded RNA (dsRNA). Each of the genome segments either encodes one of the structural proteins (VP1 to VP4, VP6 and VP7) or one of the non-structural proteins (NSP1 to NSP6) [6]. All RoVs are classified into the genus *Rotavirus* within the family *Reoviridae*. Based on genome analysis and amino acid sequence identities of VP6, rotavirus species A–J have been defined and the putative additional species K and L have recently been described. Among them, rotavirus A is by far the most medically important species, resulting in severe acute diarrhea in infants and young children worldwide [7–9]. Rotavirus vaccination is one of the most effective preventive strategies. Countries that introduced rotavirus vaccines and had high coverage rates have reported substantial declines in the incidence of rotavirus gastroenteritis [10–12].

Noroviruses (NoVs) belong to the *Caliciviridae* family and consist of non-enveloped, small RNA virus particles containing a single-stranded, positive-sense, poly-adenylated RNA genome. Human NoVs can be classified into three geno-groups and numerous genotypes within each geno-group (i.e., 9 GI, 25 GII, and 1GIV) [13]. NoVs were associated with approximately one-fifth of all diarrhea cases and more than 200,000 deaths per year in the low-income countries. Young children experienced the highest incidence of disease, while severe outcomes were most common among young children and the elderly, persisting due to the lack of an effective vaccine and specific antiviral drugs [14, 15].

Some rotavirus- and norovirus-associated pediatric AGE studies have been performed in coastal and urban areas in China, but there are few epidemiological reports from western regions due to limited technological development and poor economic conditions [16–25]. Yunnan Province (Southwest China) shares a border of 4060 km with Myanmar in the west, Laos in the south and Vietnam in the southeast of China. Kunming is the capital of the province and lies in the low latitude plateau monsoonal climate region with an annual average temperature of approximately 15 ℃. The rainy season lasts from May to September, and the annual average relative humidity is 74%. The permanent resident population composed of numerous ethnicities of Kunming was approximately 8.46 million in 2020.

As of September 2018, only one monovalent Lanzhou lamb rotavirus (LLR) vaccine (Lanzhou Institute of Biological Products, Lanzhou, China) has been licensed since 2000. The effectiveness of LLR vaccine was reported in several post-marketing studies conducted in some areas of China during 2007–2012, but the results remained controversial which was from 35.0 to 73.3% [26–28]. Significantly, one human-bovine reassortant pentavalent vaccine, RV5 (RotaTeq™, Merck & Co., Inc., Kenilworth, NJ, USA) has been provided in China since September 2018. The RotaTeq vaccine showed 79% efficacy against moderate to severe rotavirus gastroenteritis [29]. Unfortunately, rotavirus vaccination is not included in China’s national immunization program, resulting in low coverage rates due to only private market use [30].

Accordingly, the current study reports the incidence and seasonal distribution of rotavirus and norovirus as a cause of pediatric acute gastroenteritis in outpatient settings and inpatients in Yunnan Province from 2018 to 2020, aiming to provide a reference for the development of vaccine as well as to improve prevention and control of pediatric viral gastroenteritis.

## Methods

### Sample Collection

Between January 2018 and December 2020, stool samples were obtained from children under 5 years of age with acute gastroenteritis from Yan’an Hospital and Kunming Children’s Hospital. According to the National Surveillance Protocol for Viral Diarrhea (2017 version), the criteria for AGE were defined as ≥ 3 instances of loose stool or looser-than-normal stool within a 24-h period combined with significant changes to the fecal exterior, including watery or thin paste texture and the presence of mucous or < 3 stools per day with abnormal stools possibly accompanied by vomiting, abdominal pain, fever, nausea, and dehydration; the definition excluded the presence of pus or blood. Stool samples were collected immediately after patients were diagnosed with AGE and before treatment was administered. Samples sent for the detection of rotavirus or norovirus or both according to the attending physician’s discretion were evaluated retrospectively. The study was approved by the Institutional Review Board of the Kunming Children’s Hospital and Yan’an Hospital.

### Detection of Rotavirus and Norovirus

Briefly, a 10% (w/v) stool suspension of each of the samples was prepared in sterile saline at clinical laboratories of the Yan’an Hospital of Kunming city and Kunming Children’s Hospital during January 2018 to December 2020. After centrifugation at 6000 *g* for 5 min, the supernatant of the suspension was used for detection of rotavirus antigen using a Group A Rotavirus Diagnostic Kit (Colloidal Gold Device, Beijing Wantai Biologic Pharmacy Enterprise Co. Ltd, Beijing, China) according to the manufacturer’s instructions. The viral genome was extracted from the supernatants using a TIANamp Virus RNA Kit (Tiangen Biotech, Beijing, China). Determination of norovirus was performed by TaqMan-based quantitative real-time polymerase chain reaction (qRT–PCR) using a norovirus (GI, GII, and GIV) nucleic acid testing kit (Hubei Lande Medical Technology Co. Ltd, Hubei, China) on an Applied Biosystems 7500 Real-Time PCR System (Applied Biosystems, Carlsbad, CA, USA).

### Meteorological Data

For the study period, monthly temperature (minimum and maximum) and monthly relative humidity (minimum and maximum) were recorded from the Yunnan Meteorological Bureau.

### Statistical Analysis

Individual data about demography and laboratory testing results were collected by reviewing medical records, and the data were entered into a standardized database by trained clinicians using Excel (version Office 365, Microsoft). All the data were analyzed using MATLAB 2018a (USA). Statistical analyses were performed using the SPSS 18.0 (USA). Statistical method was applied according to the characteristics of the data. *P* < 0.05 was considered statistically significant.

## Results

### Description of the Study Population

Over the 3-year period, a total of 128,867 stool samples submitted for the detection of rotavirus, norovirus or both were included for analysis. The number of specimens analyzed for rotavirus was 111,070 cases, for norovirus was 17,797 cases and for mixed rotavirus/norovirus infections was 17,113 cases. Demographic parameters, including sex, age and the year of sample collection, were recorded for each patient (Table 1).

**Table 1:** Distribution of rotavirus (RoV) and norovirus (NoV) infections in children under 5 years old with acute gastroenteritis in southwestern China, 2018~2020.

	**Rotavirus**						**Norovirus**					
	Total Cases	RoV(+) No.(%)	Outpatient Cases	RoV(+) No.(%)	Inpatient Cases	RoV(+) No.(%)	Total cases	NoV(+) No.(%)	Outpatient Cases	NoV(+) No.(%)	Inpatient Cases	NoV(+) No.(%)
**Gender**												
Male	64848	15905 (24.5)	54395	14467 (26.6)	10453	1438 (13.8)	10634	2853 (26.8)	8620	2301 (26.7)	2014	552 (27.4)
Female	46222	11329 (24.5)	39152	10385 (26.5)	7070	944 (13.4)	7163	1796 (25.1)	5913	1475 (24.9)	1250	321 (25.7)
**Age (months)**												
0-6	29762	3591 (12.1)	19107	2604 (13.6)	10655	987 (9.3)	1737	244 (14.0)	1076	123 (11.4)	661	121 (18.3)
6~12	27954	7389 (26.4)	25149	6853 (27.2)	2805	536 (19.1)	5449	1331 (24.4)	4451	1051 (23.6)	998	280 (28.1)
12~18	23728	8439 (35.6)	21900	7990 (36.5)	1828	449 (24.6)	5107	1660 (32.5)	4327	1404 (32.4)	780	256 (32.8)
18~24	12551	4087 (32.6)	11657	3872 (33.2)	894	215 (24.0)	2491	770 (30.9)	2115	655 (31.0)	376	115 (30.6)
24~30	5684	1595 (28.1)	5252	1500 (28.6)	432	95 (22.0)	1167	279 (23.9)	1005	239 (23.8)	162	40 (24.7)
30~36	3659	868 (23.7)	3396	820 (24.1)	263	48 (18.3)	639	139 (21.8)	540	117 (21.7)	99	22 (22.2)
36-48	4929	918 (18.6)	4512	881 (19.5)	417	37 (8.9)	802	146 (18.2)	668	123 (18.4)	134	23 (17.2)
48-60	2803	347 (12.4)	2574	332 (12.9)	229	15 (6.6)	405	80 (19.8)	351	64 (18.2)	54	16 (29.6)
**Year**												
2018	37608	10142 (27.0)	31406	9277 (29.5)	6202	865 (13.9)	1319	418 (31.7)	1006	345 (34.3)	313	73 (23.3)
2019	41510	11253 (27.1)	35358	10257 (29.0)	6152	996 (16.2)	8281	2097 (25.3)	6915	1750 (25.3)	1366	347 (25.4)
2020	31952	5839 (18.3)	26783	5318 (19.9)	5169	521 (10.1)	8197	2134 (26.0)	6612	1681 (25.4)	1585	453 (28.6)
**Total**	111070	27234 (24.5)	93547	24852 (26.6)	17523	2382 (13.6)	17797	4649 (26.1)	14533	3776 (26.0)	3264	873 (26.7)

### Incidence of Rotavirus Infection

Rotavirus was detected in 24.5% (27,234/111,070) of cases, with a statistically significant difference in rotavirus incidence in outpatients (26.6%; 24,852/93,547) compared to hospitalized cases (13.6%; 2382/17,523; *χ*^2^ = 1300; *P* < 0.001). There was no significant difference in rotavirus detection in boys (24.5%; 15,905/64,848) compared to girls (24.5%; 11,329/46,222; *χ*^2^ = 0.004; *P* = 0.949). The annual rotavirus detection rate showed a statistically significant decline in 2020 (18.3%; 5839/31,952) compared to 2018 (27.0%; 10,142/37,608) and 2019 (27.1%; 11,253/41,510; *χ*^2^ = 945.557; *P* < 0.001). Rotavirus incidence also showed a statistically significant difference across the various age groups, ranging from a low of 12.1% (3591/29,762) in children 0–6 months to a high of 35.6% (8439/23,728) in children 12–18 months of age (*χ*^2^ = 4900; *P* < 0.001). Most of the children infected with rotavirus (86.3%; 23,506/27,234) were aged less than 2 years (Table 1).

### Incidence of Norovirus Infection

Norovirus was detected in 26.1% (4649/17,797) of patients, with 26.0% (3776/14,533) of infections in outpatients and 26.7% (873/3264) in hospitalized cases (*χ*^2^ = 0.806; *P* = 0.369). The gender ratio (male/female) in the specimens screened for norovirus was 1.48:1 (10,634/7163). A significant difference (*χ*^2^ = 6.837; *P* = 0.009) in the norovirus detection rate was observed between boys (26.8%; 2853/10,634) and girls (25.1%; 1796/7163). The annual norovirus detection rate was statistically significant higher in 2018 (31.7%; 418/1319) compared to 2019 (25.3%; 2097/8281) and 2020 (26.0%; 2134/8197; *χ*^2^ = 23.967; *P* < 0.001). As with rotavirus, norovirus incidence also showed a statistically significant difference across the various age groups, ranging from a low of 14.0% (244/1737) in children 0–6 months to a high of 32.5% (1660/5107) in children 12–18 months of age (*χ*^2^ = 320.6; *P* < 0.001). Most of the children infected with rotavirus (86.1%; 4005/4649) were aged less than 2 years (Table 1).

### Norovirus and Rotavirus Seasonality

**Fig.1:**
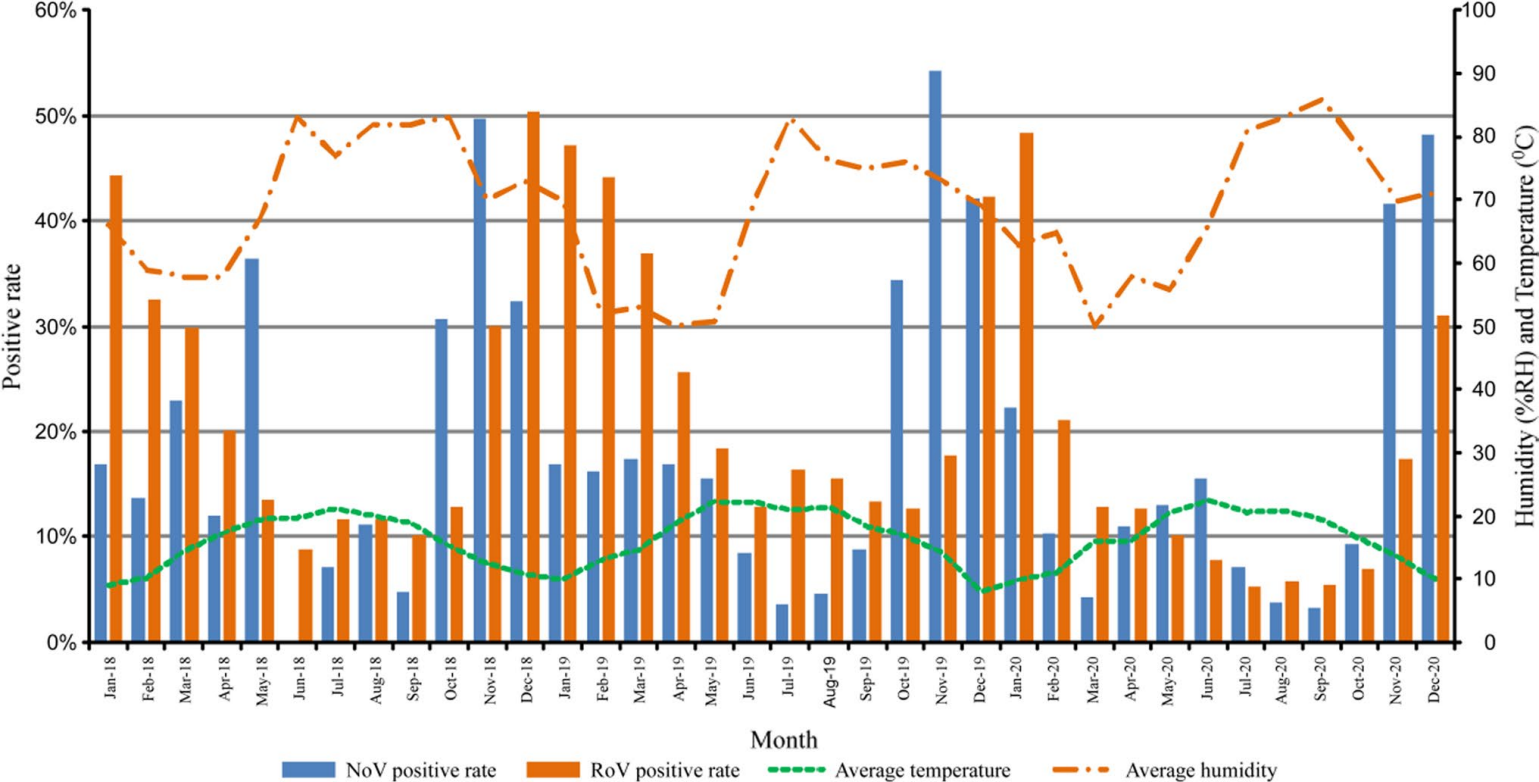
Correlation between the detection rate of norovirus and rotavirus and regional temperatures and relative humidity. Monthly distribution of norovirus and rotavirus infections from 2018 to 2020. The different trends of distribution in early 2020 may result from the COVID-19 pandemic. Both positive rates of norovirus and rotavirus were negatively correlated with the average temperature analyzed by Pearson’s chi-squared test

**Fig. 2:**
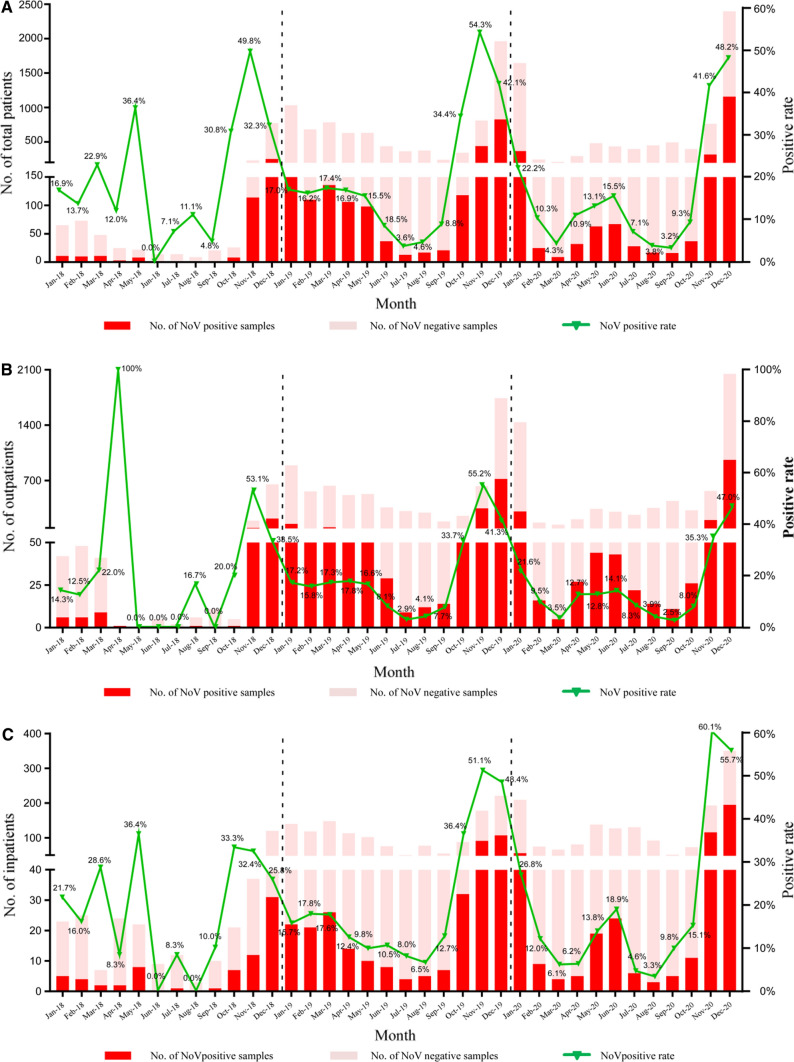
Monthly distribution of norovirus (NoV) infections in children under 5 years old with acute gastroenteritis in southwestern China, 2018–2020. Monthly distribution of norovirus infections in total patients (**A**), outpatients (**B**) and inpatients (**C**)

Norovirus seasonal distribution peaked in October to December (middle autumn to early winter) but peaked from November to December in 2020. During the warmer season from July to September, the positive rate was less than 10% (Figs. 1A and 2A). The seasonal trends of distribution in outpatients were consistent with those in inpatients during 2018–2020 (Fig. 2B, C). The positive rate of norovirus was negatively correlated with the average daily temperature (*P* = 0.042, correlation = − 0.593, Pearson’s correlation analysis). There was no correlation between the positive rate of norovirus and relative humidity (Fig. 1).

**Fig. 3:**
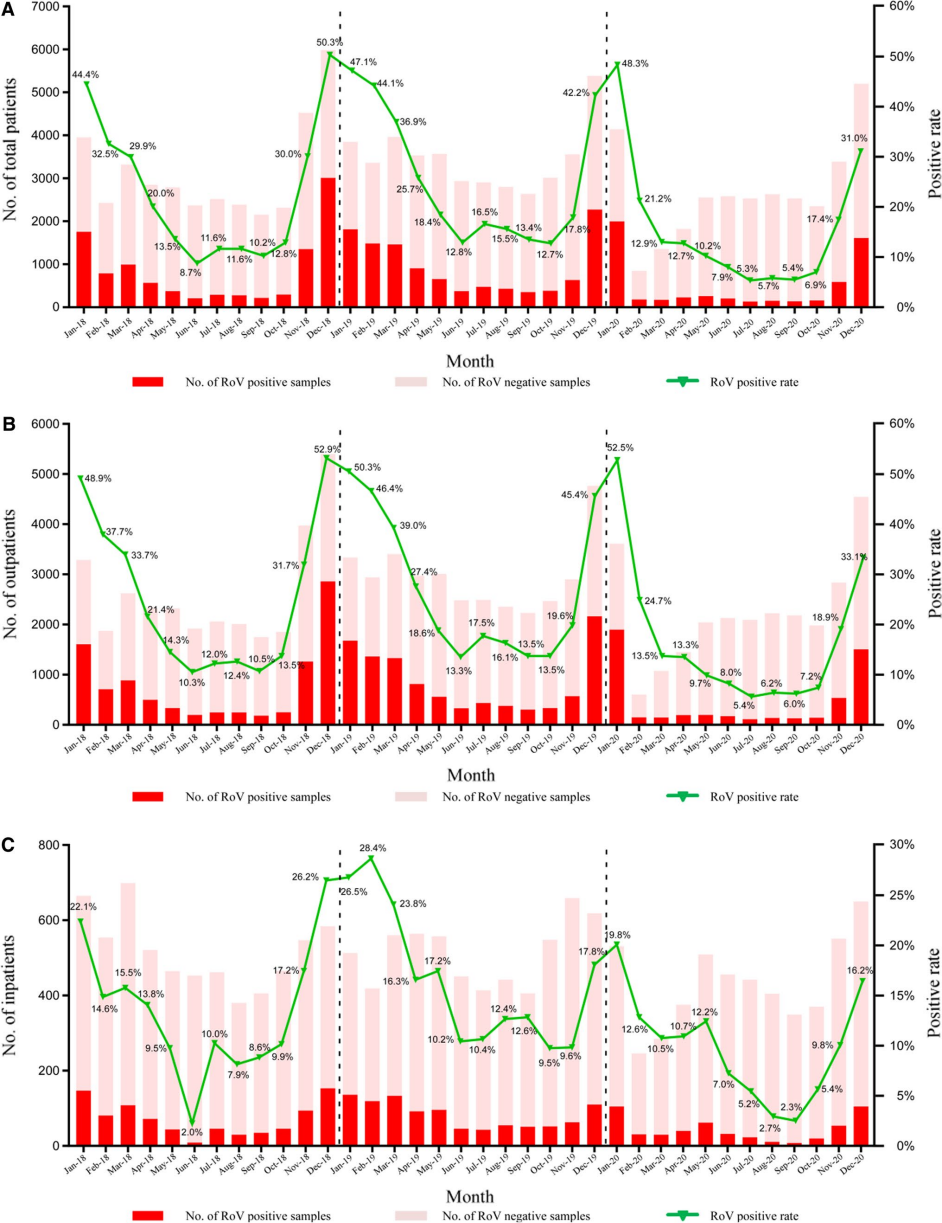
Monthly distribution of rotavirus (RoV) infections in children under 5 years old with acute gastroenteritis in Southwestern China, 2018–2020. Monthly distribution of rotavirus infections in total patients (**A**), outpatients (**B**) and inpatients (**C**)

In contrast, rotavirus seasonal distribution was more sustained, concentrated in winter (December to February) to early spring (March), with the highest in December, followed by a gradual decrease until June in each year of study, except for 2020 (Figs. 1 and 3A). Furthermore, even though the overall detection rate of rotavirus was higher in outpatients than in inpatients, the seasonal trends of rotavirus distribution in outpatients were consistent with those in inpatients during 2018–2020 (Fig. 3B, C). The relationship between the rotavirus-positive rate and temperature and humidity is shown in Fig. 3A. Pearson’s correlation analysis showed a significant correlation between the rotavirus-positive rate and average monthly temperature but not relative humidity (*P* < 0.001; correlation = − 0.916; Fig. 1).

### Rotavirus and Norovirus Infections Following the Start of the COVID-19 Pandemic

Sharp declines in children presenting for the treatment of diarrhea were noted early in 2020 with the emergence of the SARS-CoV-2 and the global pandemic, resulting in altered rotavirus and norovirus incidence in 2020 (Figs. 2A and 3A). Both norovirus and rotavirus detection rate reduced by 35–50% in February 2020 (16.2–10.3% and 44.1–21.2%, respectively; Fig. 2A and 3A). As mentioned above, rotavirus positivity rates have remained at a low level in 2020, coinciding with the onset of the COVID-19 (Coronavirus disease 2019) pandemic and mitigation factors to prevent COVID-19. However, norovirus returned to pre-pandemic levels at the end of the year.

### Incidence of Rotavirus–Norovirus Coinfections

Mixed rotavirus/norovirus infections are common in viral gastroenteritis cases and may increase the severity of diarrhea symptoms [16]. During the study period, a total of 17,113 specimens submitted for the detection of both rotavirus and norovirus were included for co-infection analysis (Table 2). The detection rate of single rotavirus infection was 27.6% (4718/17,113) and single norovirus infection was 21.1% (3616/17,113). Coinfections of rotavirus/norovirus were identified in 859 specimens (5.0%; 859/17,113), with a statistically significant difference in coinfections incidence in outpatients (5.4%; 762/14,187) compared to hospitalized cases (3.3%; 97/2926; *χ*^2^ = 21.507; *P* < 0.001). There was statistically significant difference in coinfections detection rate in boys (5.3%; 545/10,212) compared to girls (4.6%; 314/6901; *χ*^2^ = 5.347; *P* = 0.021). Moreover, the annual coinfections detection rate showed a statistically significant decline in 2020 (3.9%; 304/7851) compared to 2018 (8.1%; 109/1340) and 2019 (5.6%; 446/7922; *χ*^2^ = 55.139; *P* < 0.001) (Table 1).

**Table 2:** Rotavirus (RoV) and norovirus (NoV) co-infection in children under 5 years old with acute gastroenteritis in southwestern China, 2018–2020

	Patient no.	Mono-RoV( +) No. (%)	Mono-NoV( +) No. (%)	Co-infection No. (%)
Patients				
Outpatients	14,187	4151 (29.3)	2906 (20.5)	762 (5.4)
Inpatients	2926	567 (19.4)	710 (24.3)	97 (3.3)
Gender				
Male	10,212	2824 (27.7)	2190 (21.4)	545 (5.3)
Female	6901	1894 (27.4)	1426 (20.7)	314 (4.6)
Year				
2018	1340	540 (40.3)	294 (21.9)	109 (8.1)
2019	7922	2321 (29.3)	1552 (19.6)	446 (5.6)
2020	7851	1857 (23.7)	1770 (22.5)	304 (3.9)
Total	17,113	4718 (27.6)	3616 (21.1)	859 (5.0)

**Fig. 4:**
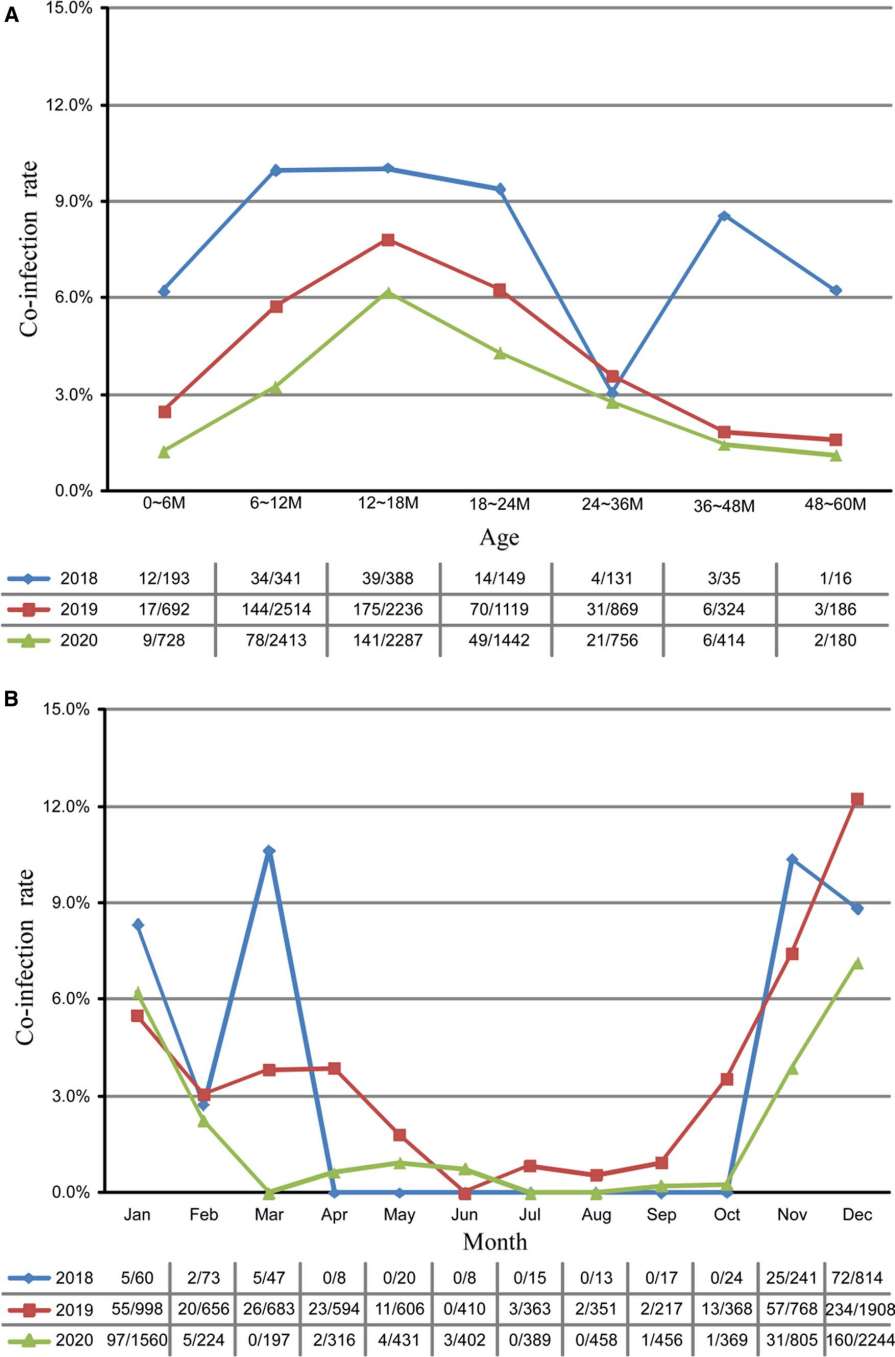
Age and seasonal distribution of rotavirus and norovirus co-infected cases in children under 5 years old with acute gastroenteritis in southwestern China, 2018–2020. Age (**A**) and seasonal (**B**) distribution

Coinfections of rotavirus/norovirus also showed a statistically significant difference across the various age groups, ranging from a low of 1.6% (6/382) in children 48–60 months to a high of 7.2% (355/4911) in children 12–18 months of age (*χ*^2^ = 113.06, *P* < 0.001). Most of the coinfections were identified in children aged less than 2 years (91.0%; 782/859) (Fig. 4A). In line with the single infections, coinfections were concentrated in November to March, with the highest in December. During the warmer season from June to September, the positive rate was less than 1% (Fig. 4B).

## Discussion

In this study, a 3-year systematic investigation of rotavirus and norovirus infections was carried out in children under 5 years of age with AGE in Southwest China. While rotavirus and norovirus were responsible for roughly a quarter of diarrhea cases in this study, approximately 50% of the specimens were negative after screening suggesting that other enteric viruses, such as adenovirus and astrovirus, as well as bacterial and parasites pathogens may also contribute to the diarrheal disease burden [31, 32].

The detection rates of rotavirus in AGE cases in the current study (24.5%) were equal to those of countries had introduced rotavirus vaccine in their national immunization programs, where rotavirus was detected in 23.0% of admissions for AGE annually. However, the incidence of rotavirus in this study was lower than those vaccines that have not been introduced yet, where rotavirus was detected in 38.0% of admissions for AGE [33]. This prevalence is remarkable given that vaccine coverage rate in this region is quite low due to only private market use of rotavirus vaccines. Thus, vaccine use is probably not the main reason for the lower rotavirus detection rate in Southwest China. Moreover, we found that rotavirus-associated AGE in outpatient settings (26.6%) was approximately 2 times more common than in inpatient settings (13.6%) in Southwestern China. However, these results were different from some studies conducted in other regions of China, which showed that rotavirus positivity was greater among inpatients than outpatients [23, 34]. This may be because rotavirus testing has been fully incorporated in routine clinical detection in outpatient settings in our surveillance hospitals since 2017. According to our criteria for AGE, cases were included not only with abnormal stools but also with vomiting, abdominal pain, fever, nausea, and dehydration; hence, both asymptomatic and symptomatic pediatric patients were included in our outpatient settings. In addition, parents now tend to take their children to hospitals when they have less severe diarrhea, which lowers the risk of hospitalization. Several studies in China showed decreasing rates of hospitalization of children due to diarrhea [23, 34].

It is worth noting that norovirus testing was not a routine clinical detection method in Yunnan Province due to the slow development of technology until 2018. Thus, the current study first reported that the overall detection rate of norovirus was 26.1% in children under five years old from 2018 to 2020 in Southwest China. The detection rate was similar to some hospital-based sentinel surveillances for norovirus diarrhea in other regions of China, while was higher than the globally norovirus incidence (about 20%) [14, 18, 24]. Regional differences in norovirus in China are also noteworthy, and norovirus was more prevalent in North China than in South China [21, 24]. Unlike rotavirus, the incidence of norovirus infections was similar between outpatients and inpatients in other regions of China and our study [24]. However, the results from a meta-analysis of the global prevalence of norovirus showed that NoVs were more frequent in outpatients than in inpatient children [14].

In the current study, although the enrolled patients were under 5 years of age, the vast majority (approximately 80%) of clinical infections occurred under the age of 2 years. The burden of both rotavirus- and norovirus-associated AGE was higher in this age group, especially in patients aged 12–18 months, followed by children aged 18–24 months. The same age distribution has been reported in previously published studies [21, 35]. Meanwhile, the incidence of viruses was low in infants aged 0–6 months. It is generally believed that maternal antibodies could be a protective factor for children early in their life during the breastfeeding period [36, 37]. In addition, rotavirus and norovirus infections might result in protective immunity against reinfection after 2 years of age. Several recently published modeling studies have estimated that immunity against norovirus lasts from 4 to 8 years post infection [38].

Mixed rotavirus/norovirus infections are common in viral gastroenteritis cases. In the current study, the co-infection of rotavirus and norovirus was found to be 5.0%, which was higher than some previous studies [16, 39]. The distribution of these co-infection cases was similar to that of either norovirus or rotavirus in terms of age and seasonality, which is consistent with data reported by Mikounou Louya et al. [40]. The yearly co-infection rate showed a significant steady decrease during the study years, which may be related to the decreasing regional detection rate of rotavirus. From 2018 to 2020, the detection rate of rotavirus in the same population decreased from 48.4 to 27.5% (Table 2).

With regard to seasonality, both viruses were detected throughout the year. Although the distribution of norovirus-associated AGE varies between regions, it is believed to be associated with heavy rainfall, low temperature, and high humidity in mathematical models and some previous studies. In general, more norovirus infections are detected during colder months [19, 39, 41]. Our study demonstrated that most norovirus infections were detected in October to December (middle autumn to early winter), while another study found that the seasonal peak of norovirus occurred between late summer and fall in the southeastern areas of Shanghai and Hangzhou and southwestern areas such as Chongqing in China [24]. Furthermore, during the warmer season from July to September, the positive rate was less than 10% in our study. However, in some other areas, norovirus-associated diarrhea had a summer peak or no apparent seasonal peak, which may be connected with an increase in contaminated water and food or other unknown reasons [24]. The peak of rotavirus infections occurred in autumn and winter in different countries [42]. In the present study, rotavirus infections were more sustained, concentrated in winter (December to February) to early spring (March). The peak of norovirus infections appeared earlier than that of rotavirus infections in our study. However, the peak of norovirus infections obtained from other studies seemed to overlap with that of rotavirus infections [24, 43]. Changes in environmental conditions, such as humidity and temperature cycles, are associated with the seasonality of infectious diseases [44]. Based on our findings, both norovirus and rotavirus infections were negatively correlated with the ambient temperature but not the humidity. This results that that low temperatures may be associated with an increase in virus incidence indicated a possibly favorable physical environment for the virions. In addition, more indoor socializing and close contact during winter months may result in high chance of virus transmission.

In the present study, both the numbers of children seeking medical care with acute gastroenteritis and virus incidence decreased sharply in February 2020 under China's prevention and control measures response on January 25, 2020 for SARS-CoV-2. Remarkably, these health safety measures not only curb the spread of COVID-19 and also effectively control the transmission of other infectious diseases, such as respiratory, influenza, and mycoplasma infections [45–47]. The detection rate of rotavirus from February to December 2020 was obviously lower than that in 2018 and 2019. However, our study showed that norovirus incidence has already returned to the pre-pandemic levels at the end of the year 2020. It is reasonable to presume that COVID-19 health safety measures, including stay-at-home orders, social distancing, and hand hygiene, are temporally associated with a decline in viruses transmission. To address this, more studies are needed to investigate the effect of protective measures on virus infection before and after the COVID-19 pandemic.

There were several limitations with our study. We did not compare clinical features among the patients but retrieved only the demographic data and epidemiologic data of the patients, so the disease severity associated with viral infections in children with acute gastroenteritis was not available. Second, this study was only conducted in two local hospitals, so the results may not be applicable to the general population. Last, genotyping of rotovirus and norovirus strains were not characterized in this study and thus we were unable to provide the molecular epidemiology during the study period in southwestern China.

## Conclusion

In conclusion, this study has, for the first time, described the considerably high burden of norovirus and rotavirus gastroenteritis in children under 5 years of age with acute gastroenteritis before introducing an obligatory rotavirus vaccine to the national immunization program in Southwest China. To provide a better understanding of pediatric viral gastroenteritis and assess targeted prevention strategies effectively, long-term continuation and collection of active surveillance data should be conducted by future epidemiological studies.

## Data Availability

The data that support the findings of this study are available on request from the corresponding author.

## Abbreviations

AGE: Acute gastroenteritis
dsRNA: Double-stranded RNA
RoVs: Rotaviruses
NoVs: Noroviruses
LLR: Lanzhou lamb rotavirus
qRT–PCR: Quantitative real-time polymerase chain reaction
COVID-19: Coronavirus disease 2019
